# Supplementary material for: Anti-Tumor Effects of a Penetratin Peptide Targeting Transcription of E2F-1, 2 and 3a Is Enhanced When Used in Combination with Pemetrexed or Cisplatin
Source: Cancers (Basel). 2021 Feb 26;13(5):972. doi: 10.3390/cancers13050972 (PMC7956530; doi:10.3390/cancers13050972)
Supplement: Supplementary file 1 [file cancers-13-00972-s001.pdf]

cancers

MDPI

Article

Anti-Tumor Effects of a Penetratin Peptide Targeting Transcription of E2F-1, 2 and 3a Is Enhanced When Used in Combination with Pemetrexed or Cisplatin

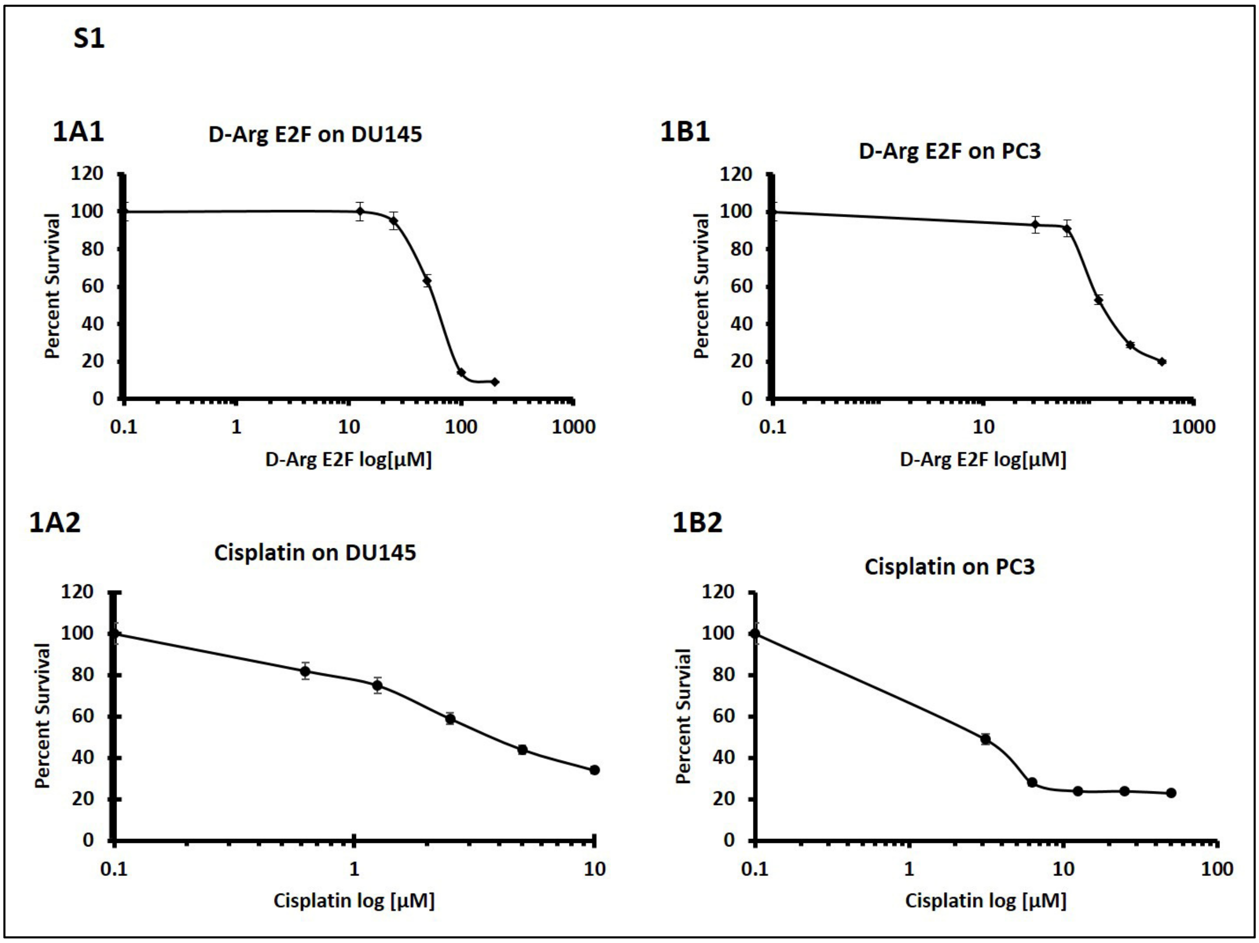

Figure S1. Cytotoxicity effect of D-Arg PEP and cisplatin on prostate cancer cell lines, DU145 (1A1-1A2) and PC-3 (1B1-1B2). In this assay, 5000 cells per well were plated in a 96 well plate on day zero in RPMI media containing 10% FBS. After 24 hours, D-Arg PEP or cisplatin was added as per the concentration shown for individual drugs. The cell viability was assessed at 48 hours of treatment by measuring the absorption at 490nm using the MTS tetrazolium Promega CellTitre 96® Aqueous One Solution according to the manufacturer's instructions. IC_{50} value was determined using the non-linear regression curve fit of the graphs drawn by GraphPad Prism. Values shown are mean ± S.E. All experiments were performed in triplicate, and all experiments were repeated at least three times.

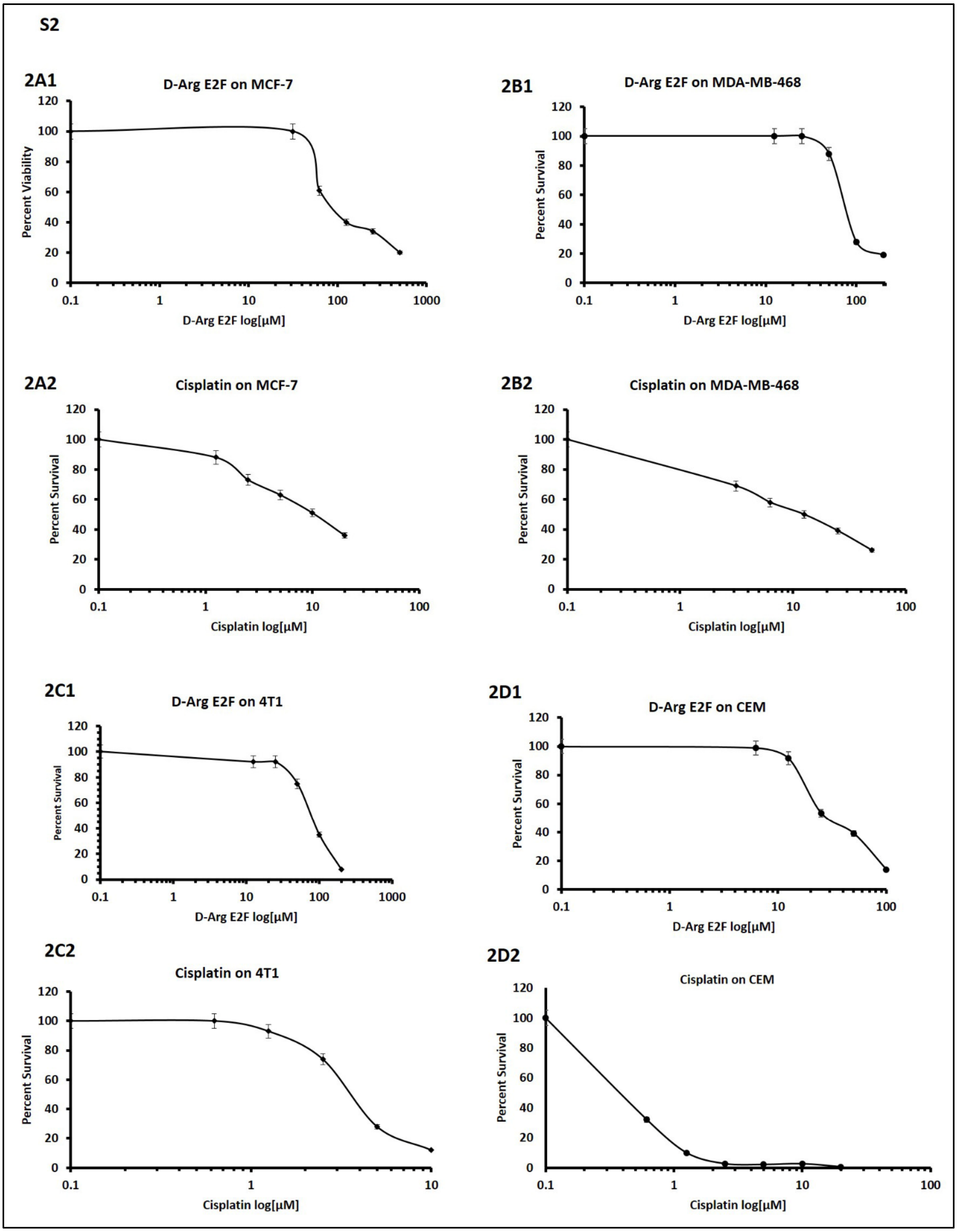

Figure S2. Cytotoxicity effect of D-Arg PEP and cisplatin on breast cancer cell lines, MCF7 (2A1-2A2), MDA-MB-468 (2B1-2B2), and 4T1 (2C1-2C2) and lymphoma CCRF-CEM (2D1-2D2). In this assay, 5000 cells per well were plated in a 96 well plate on day zero in RPMI media containing 10% FBS. After 24 hours, D-Arg PEP or cisplatin was added as per the concentration shown for individual drugs. The cell viability was assessed at 48 hours of treatment by measuring the absorption at 490nm using the MTS tetrazolium Promega CellTitre 96® Aqueous One Solution according to the manufacturer's instructions. 5000 CCRF-CEM cells (suspension) were plated in a 12-well plate in RPMI 1640 media supplemented with 10% FBS. After 24 hours, the drug was added and incubated for 48h. To assess cell viability, the cells with or without drug

treatment were collected and cell viability was determined using the Vi-CELL™ Series Cell Viability Analyzer (Beckman Coulter, Carlsbad, CA). IC_{50} value was determined using the non-linear regression curve fit of the graphs drawn by GraphPad Prism. Values shown are mean ± S.E. All experiments were performed in triplicate, and all experiments were repeated at least three times.

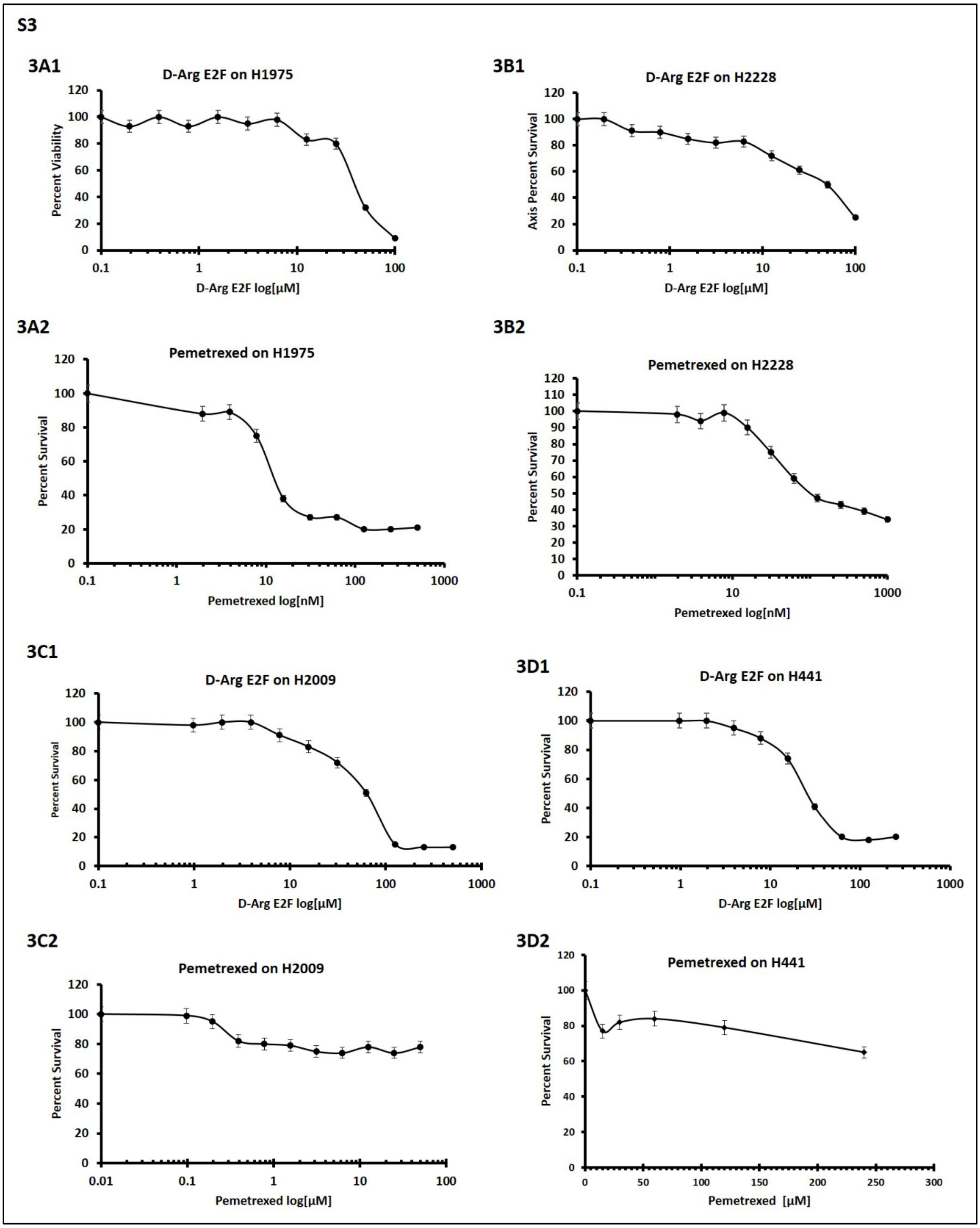

Figure S3. Cytotoxicity effect of D-Arg PEP and Pemetrexed on non-small cell lung cancer (NSCLC) cell lines; H1975 (3A1-3A2), H2228 (3B1-3B2), H2009 (3C1-3C2) and H441 (3D1-3D2). In this assay, 5000 cells per well were plated in a 96 well plate on day zero in RPMI media containing 10% FBS. After 24 hours, D-Arg PEP or pemtrexed was added as per the

concentration shown for individual drugs. The cell viability was assessed at 48 hours of treatment by measuring the absorption at 490nm using the MTS tetrazolium Promega CellTitre 96® Aqueous One Solution according to the manufacturer's instructions. IC_{50} value was determined using the non-linear regression curve fit of the graphs drawn by GraphPad Prism. Values shown are mean ± S.E. All experiments were performed in triplicate, and all experiments were repeated at least three times.

S4A1

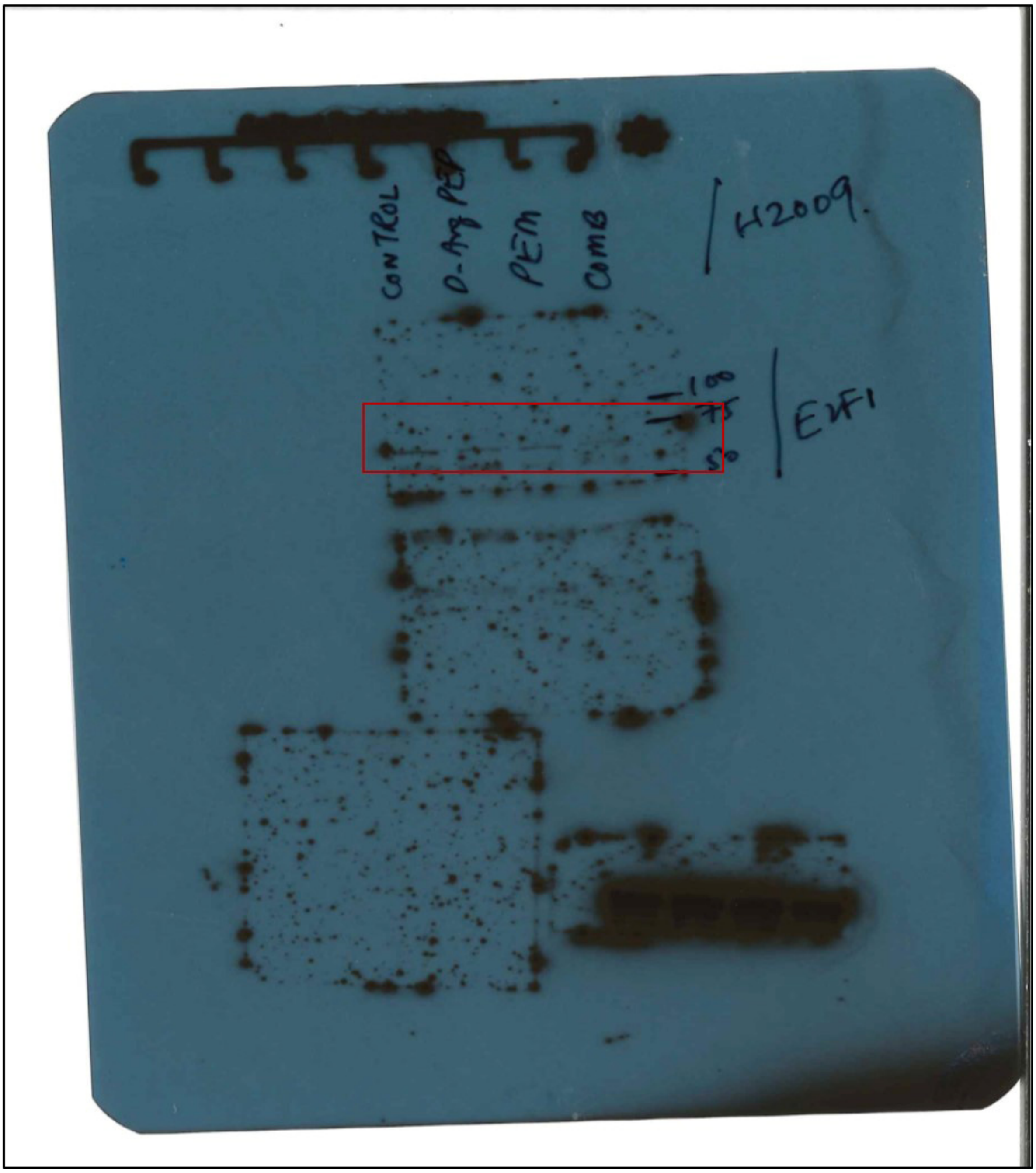

S4A2

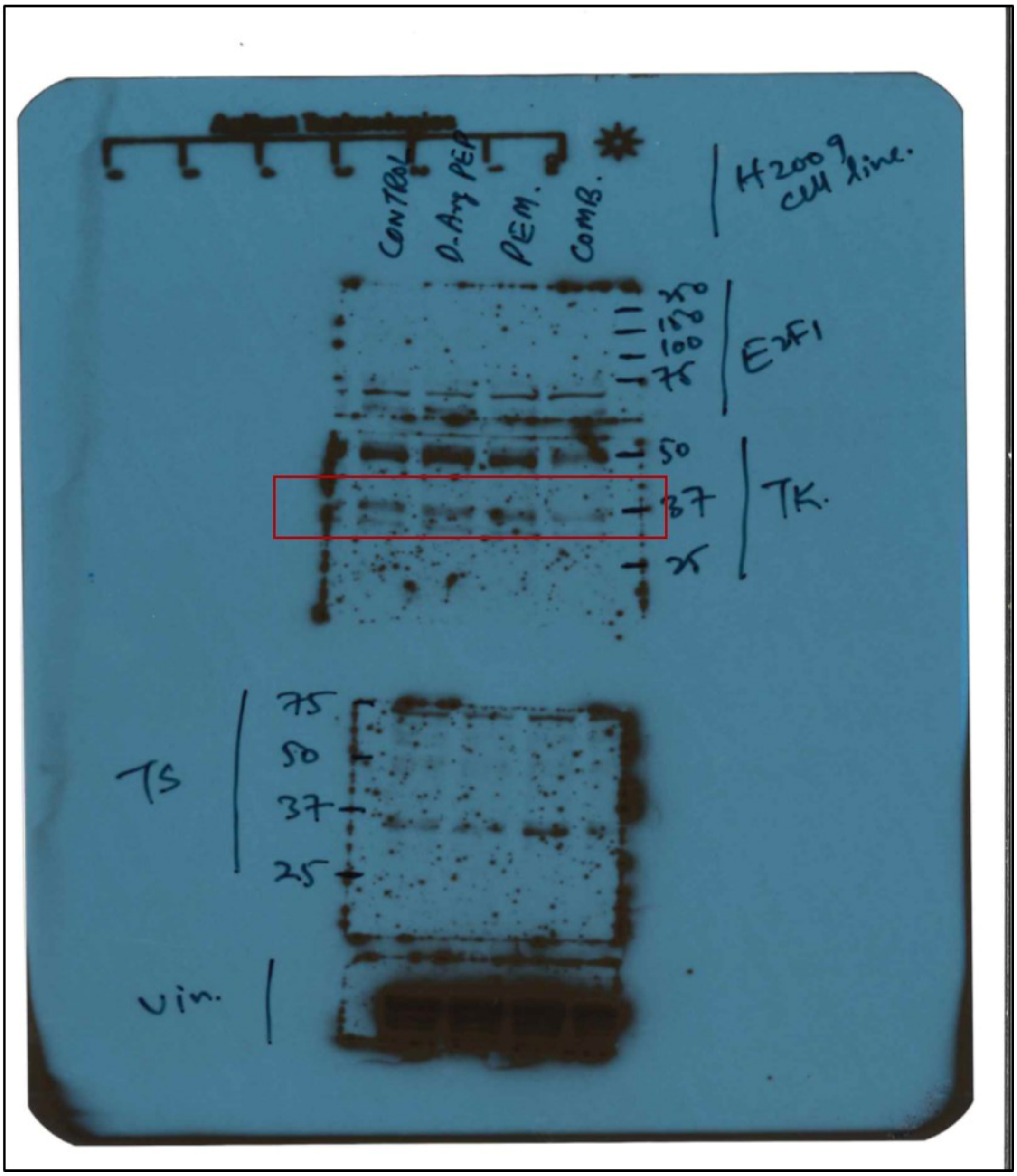
CONTROL
D-Arg PEP
PEM.
COMB.
H2009 cell line.
250
150
100
75
E2F1
50
37
25
TK.
75
50
37
25
TS
vin.

S4A3

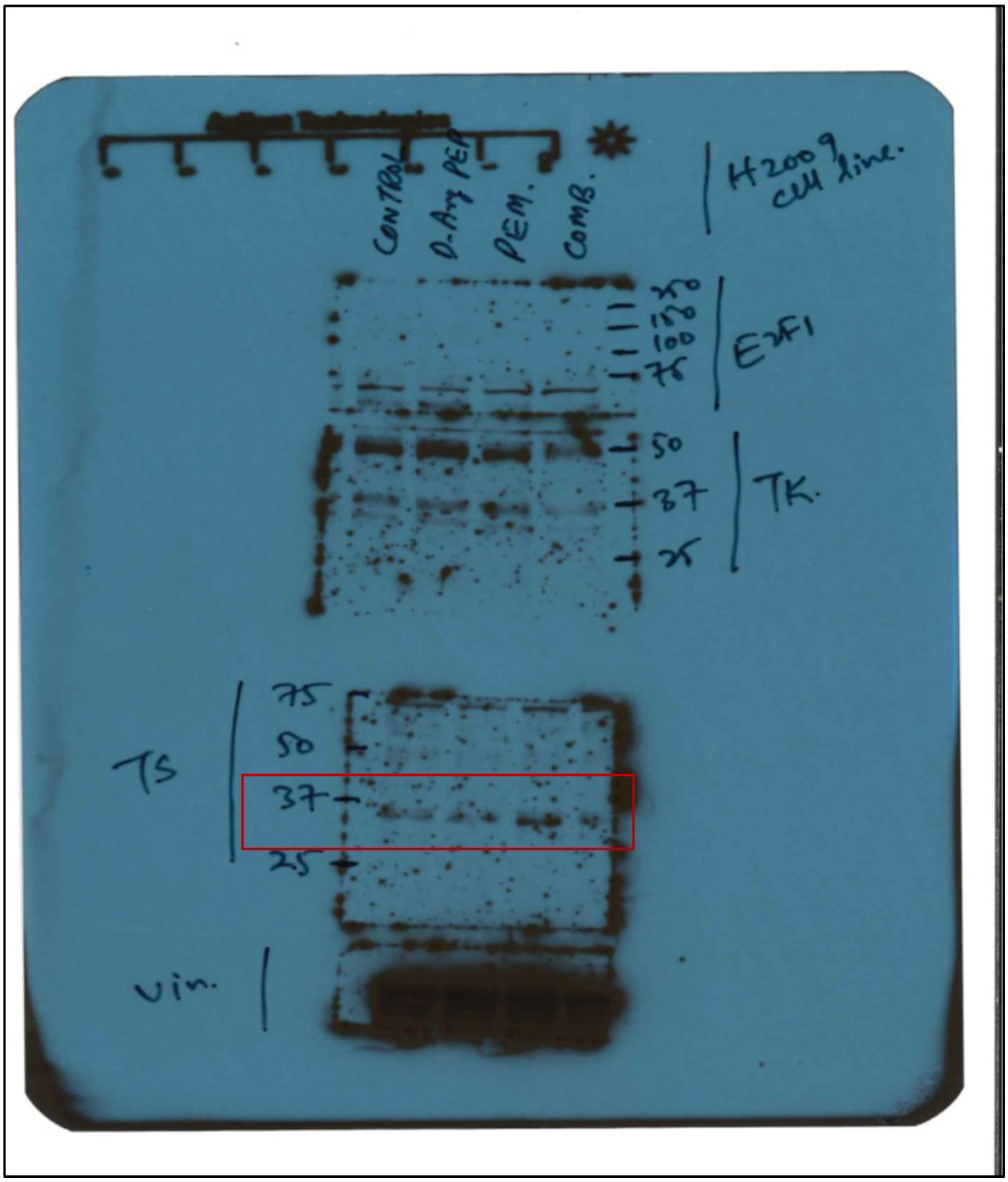
H2009 cell line.
CONTROL
PEM.
COMB.
E2F1
50
37
TK.
75
50
TS
37
25
Vin.

S4A4

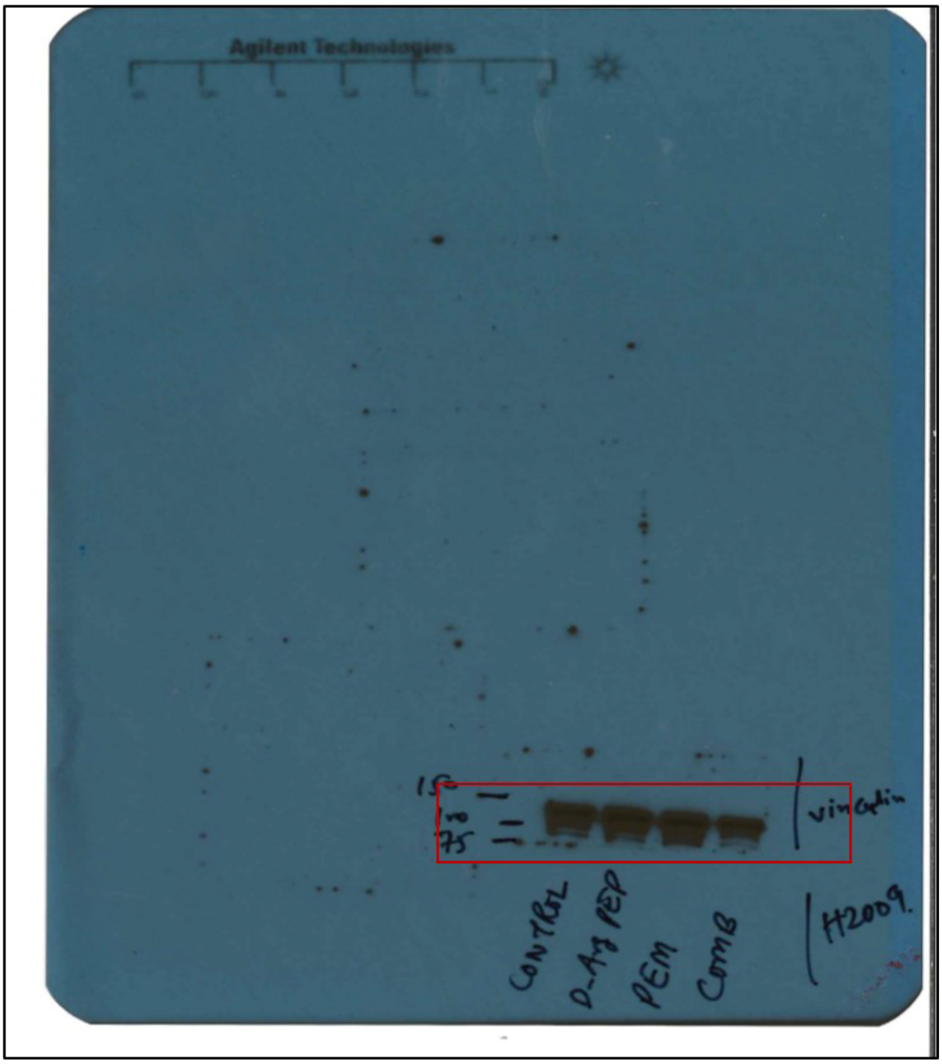

S4B1

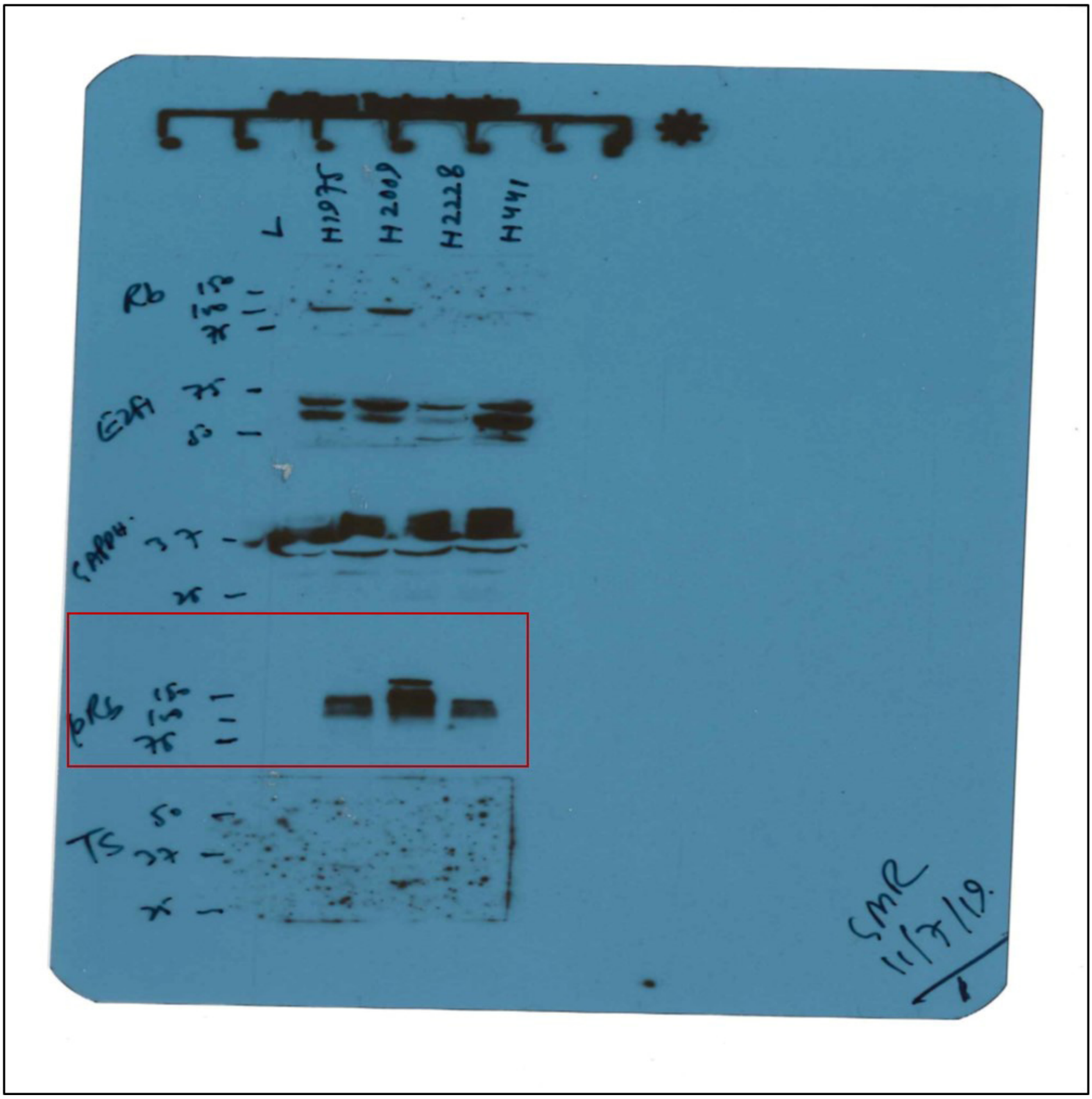

S4B2

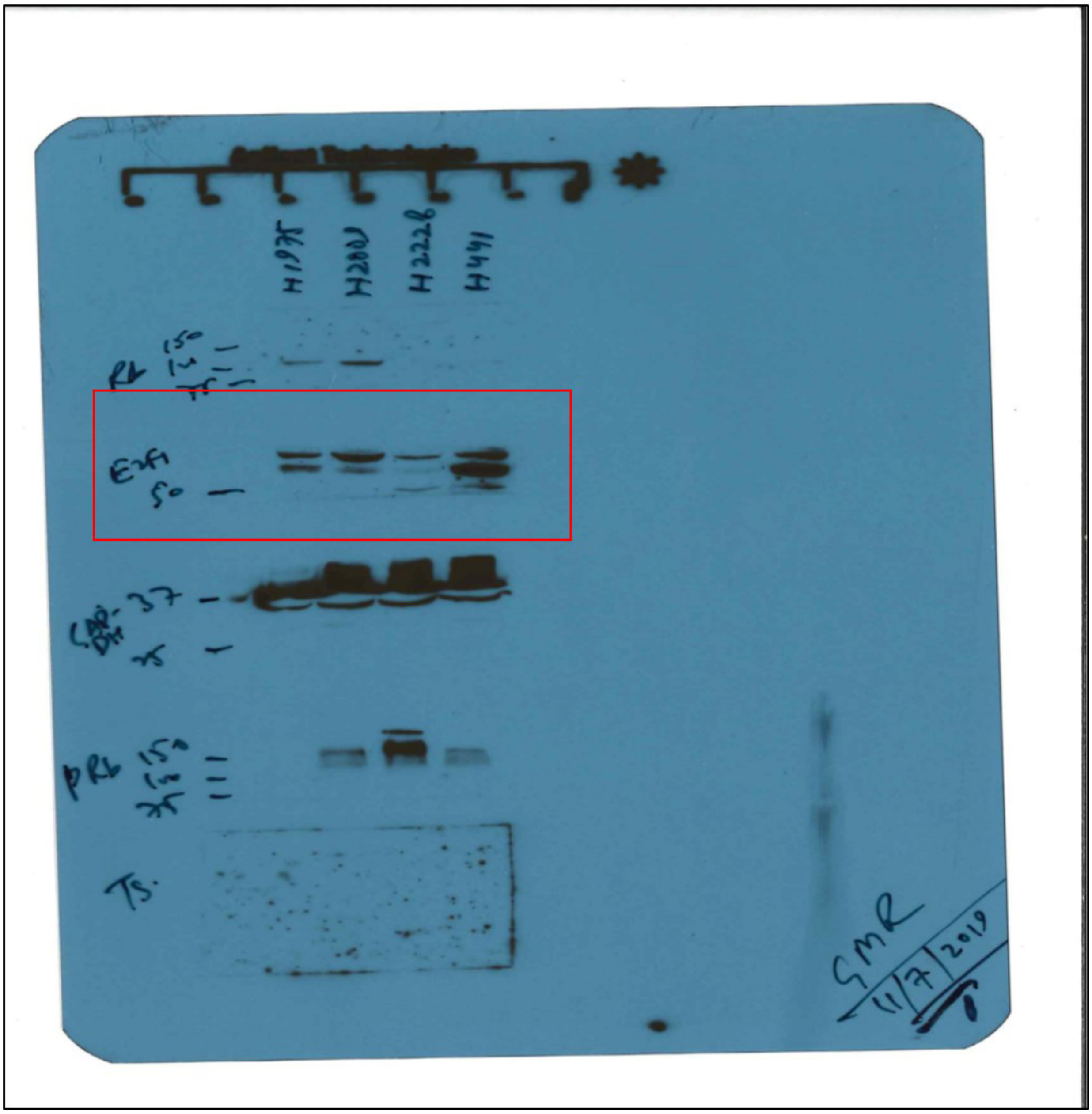

S4B3

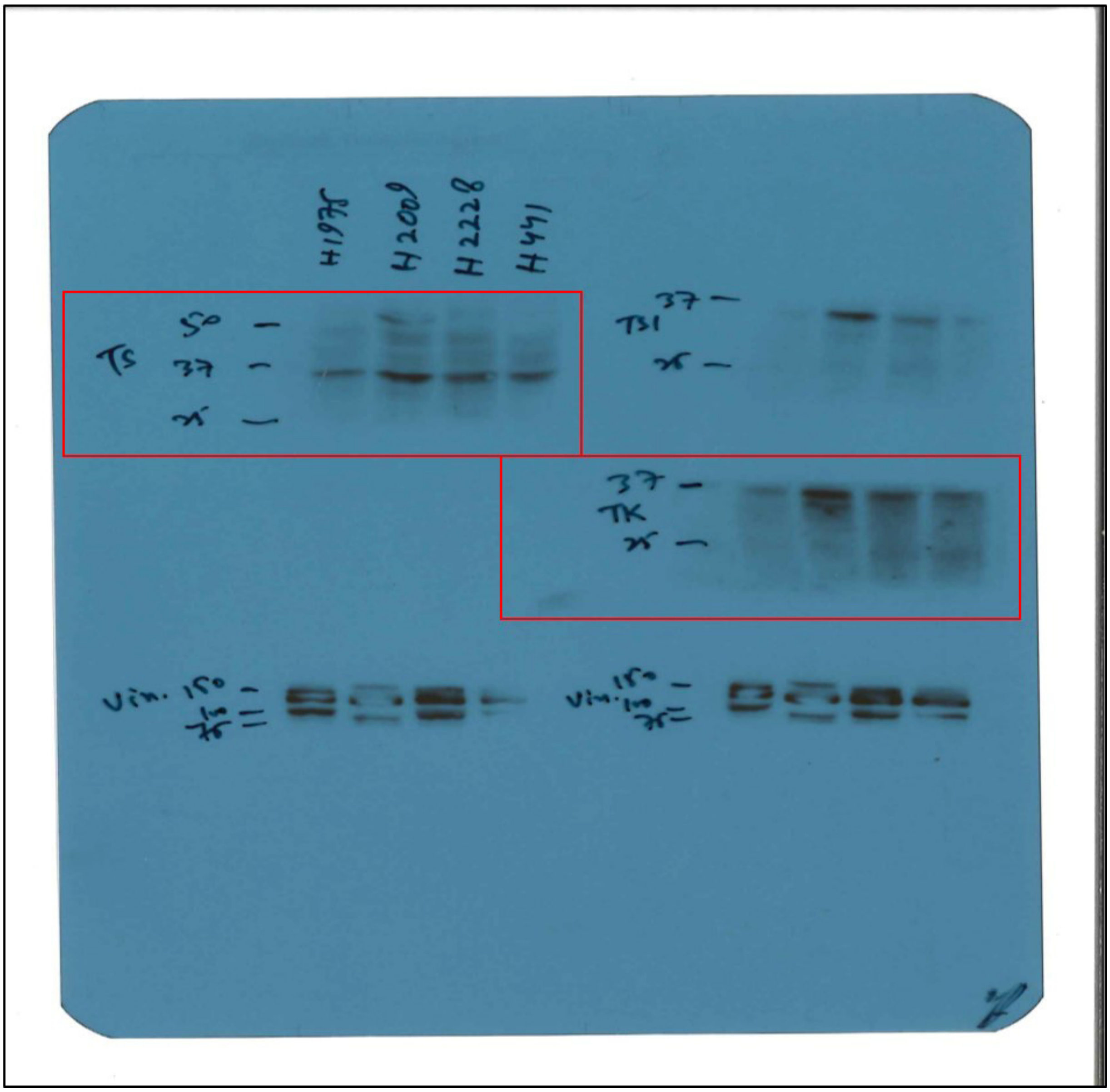

S4B4

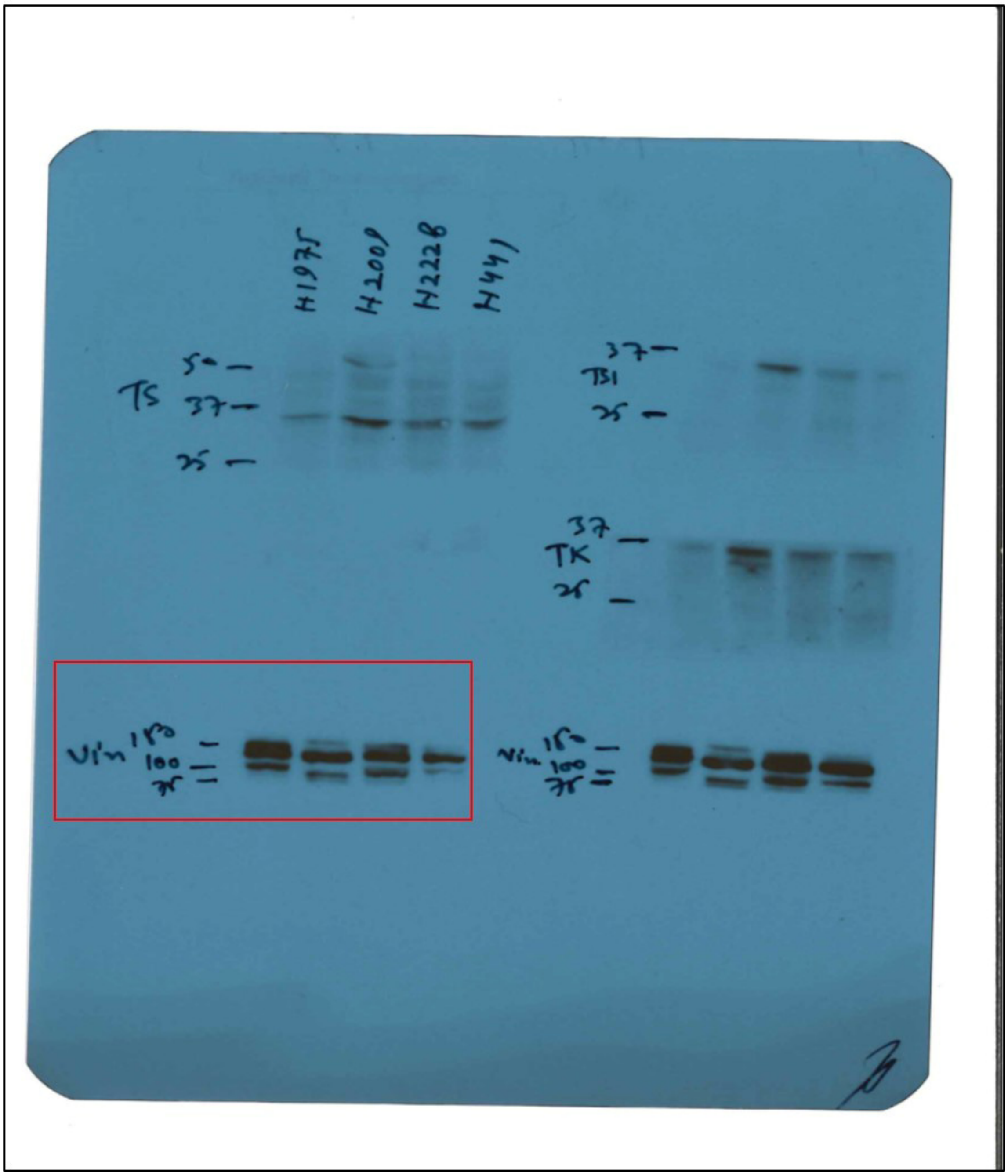

Figure S4. Uncropped western blots of the H2009 cell line after 48h treatment with IC_{50} concentration of either D-Arg PEP, or pemetrexed or combination (Figure 5F) and probed for: E2F-1 (**S4A1**), TK (**S4A2**), TS (**S4A3**) and Vinculin (**S4A4**). Uncropped western blots of the NSCLC cell lines (H1975, H2009, H2228 and H441) probed for: phosphorylated retinoblastoma (pRb) (**S4B1**), E2F-1 (**S4B2**), TS and TK (**S4B3**) and vinculin (**S4B4**).

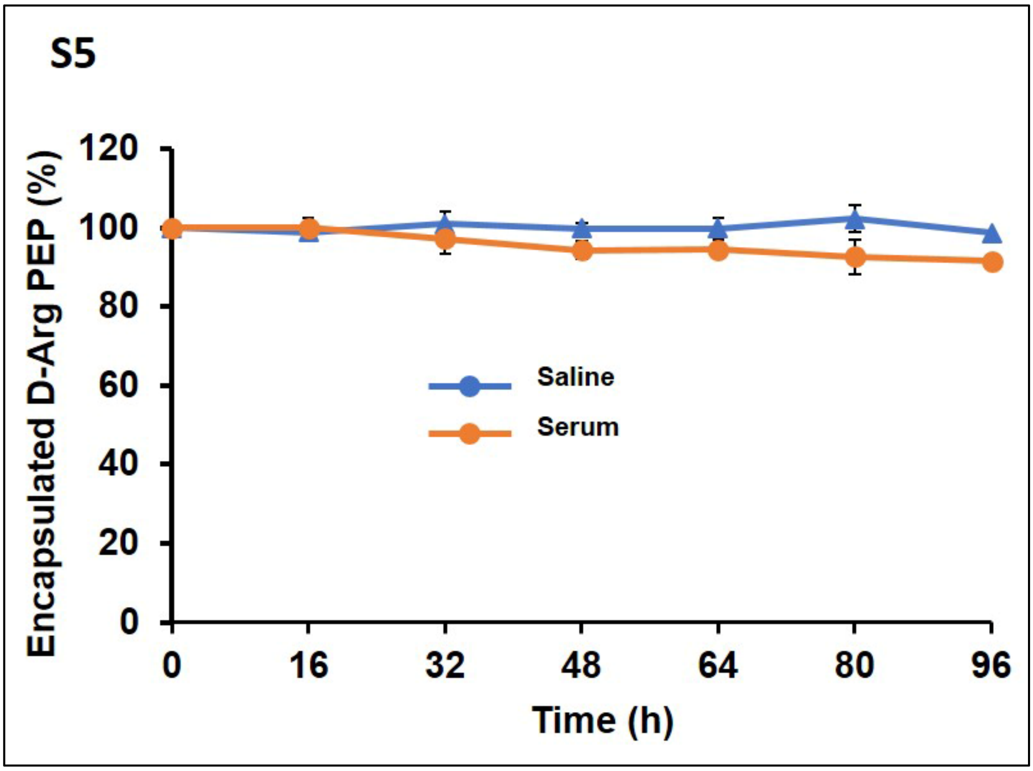

Figure 5. Release of D-Arg PEP from PEGylated liposomes. Means ± SD are shown.

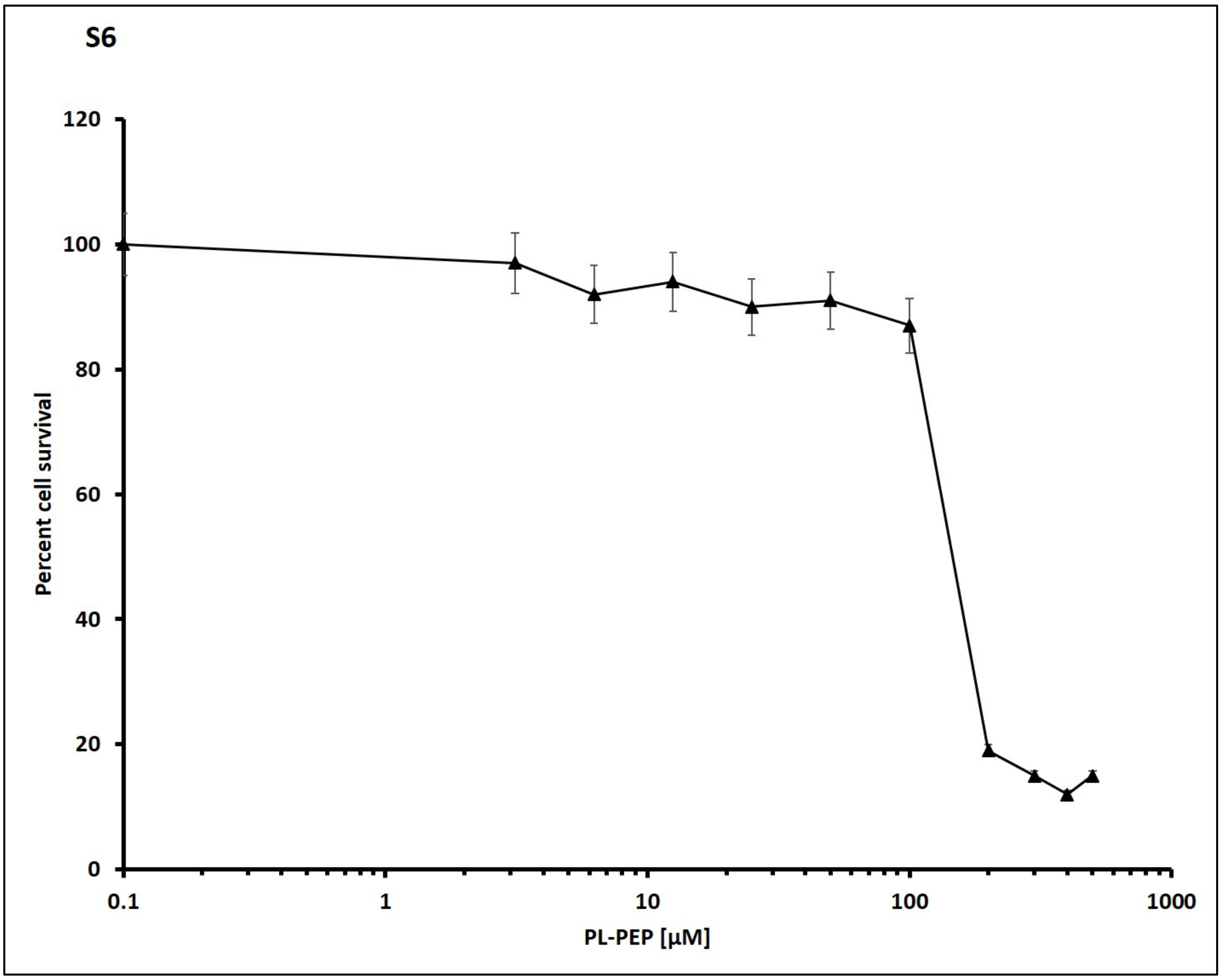

Figure S6. Effect of PEGylated liposomal D-Arg PEP (PL-PEP) against the H2009 cell line. In this assay, 5000 cells per well were plated in a 96 well plate on day zero in RPMI media containing 10% FBS. After 24 hours, PL-PEP was added as per the concentration shown. The cell viability was assessed at 48 hours of treatment by measuring the absorption at 490nm using the MTS tetrazolium Promega CellTitre 96® Aqueous One Solution according to the manufacturer's instructions. IC_{50} value was determined using the non-linear regression curve fit of the graphs drawn by GraphPad Prism. Values shown are mean ± S.E. All experiments were performed in triplicate, and all experiments were repeated at least three times.

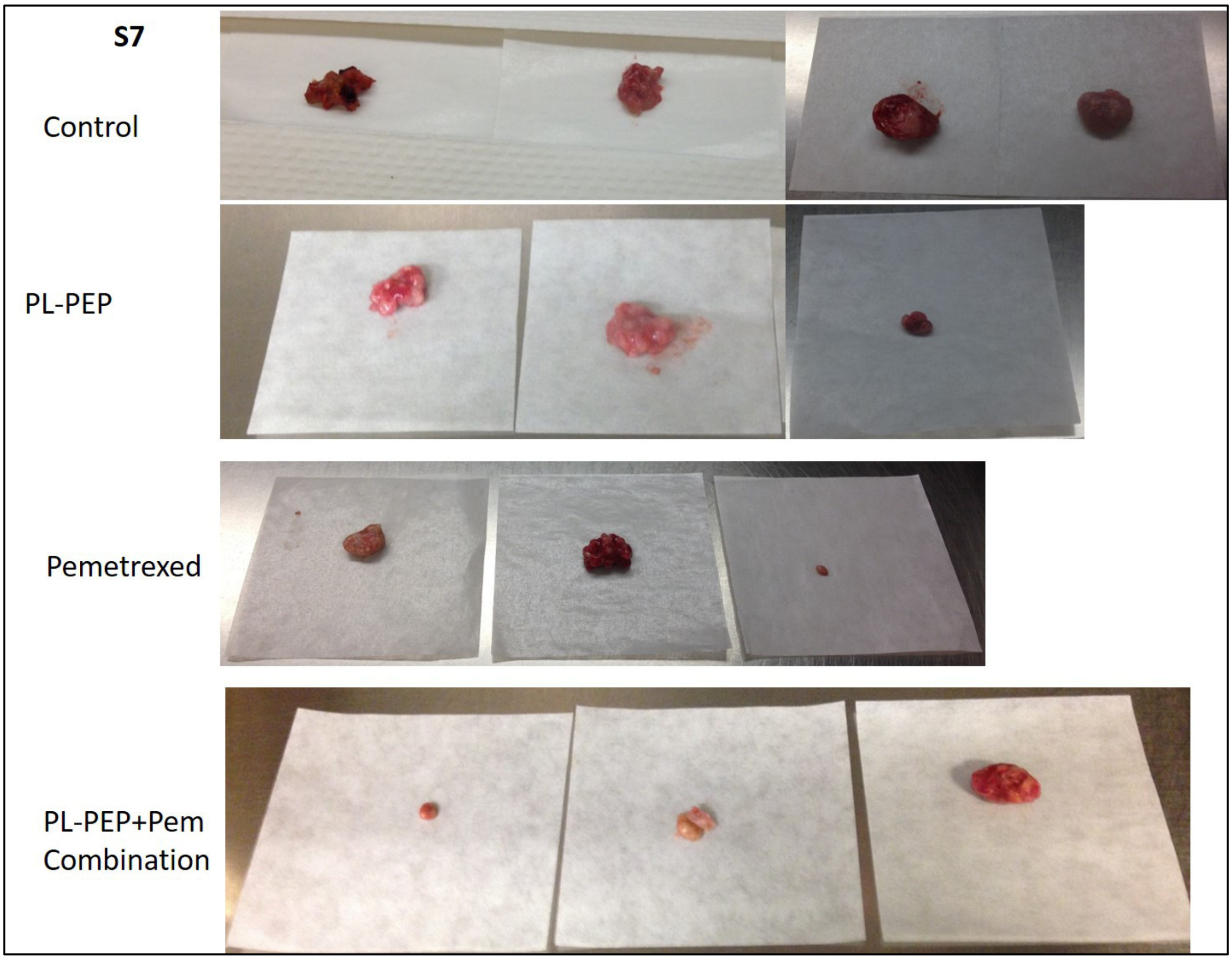

Figure S7. Tumors extracted from the H2009 xenograft study. Mice were treated with the PEGylated liposomal D-Arg PEP (PL-PEP) or pemetrexed or combination as shown in figure 7.
